# Comparison of effects of a single dose of MHYOSPHERE**®** PCV ID with three commercial porcine vaccine associations against *Mycoplasma hyopneumoniae (Mhyo)* and porcine circovirus type 2 (PCV2) on piglet growth during the nursery period under field conditions

**DOI:** 10.1007/s11259-022-09971-y

**Published:** 2022-07-13

**Authors:** Ainhoa Puig, Ignacio Bernal, David Sabaté, Isaac Ballarà, Jordi Montané, Lorena Nodar, Daniel Angelats, Ramon Jordà

**Affiliations:** 1 R&D Clinical Development, HIPRA SCIENTIFIC, S.L.U, Avda. la Selva 135, 17170 Amer, Girona, Spain; 2 Swine Business Unit, LABORATORIOS HIPRA, S.A., 17170 Amer, Girona, Spain; 3 R&D Biologicals, HIPRA SCIENTIFIC, S.L.U, 17170 Amer, Girona, Spain; 4 HIPRASTATS. LABORATORIOS HIPRA, S.A., 17170 Amer, Girona, Spain

**Keywords:** *Mycoplasma hyopneumoniae*, Porcine circovirus type 2, Welfare, Growth performance, Safety, Intradermal

## Abstract

Pigs routinely undergo stressful vaccination procedures, which are often unavoidable given the unavailability of safer alternatives, challenging animal welfare. The available vaccines for *Mycoplasma hyopneumoniae* (Mhyo) or Porcine circovirus type 2 (PCV2) are mostly administered intramuscularly in association to prevent Porcine respiratory disease complex (PRDC). MHYOSPHERE® PCV ID is the first vaccine protecting from both agents by intradermal route. This randomized, blind-field trial aimed to compare the effects of MHYOSPHERE® PCV ID with those of three different intramuscular associations of commercially available vaccines. A total of 7072 21-day-old piglets from 12 consecutive batches in one farm were randomly vaccinated with MHYOSPHERE® PCV ID (G1) or Ingelvac CircoFLEX® + Hyogen® (G2), Porcilis® PCV + M + PAC® (G3), and Porcilis® PCV + Hyogen® (G4). Growth performance during the nursery period and adverse reactions (ARs) after vaccine administration were monitored. Average Daily Weight Gain (ADWG) during the first 7 days post-weaning in G1 was 10.92, 3.03, and 20.08 g/day higher than in G2, G3, and G4, respectively, and 0.65, 4.06, and 9.58 g/day higher than in G2, G3, and G4 during the entire nursery period, respectively. G1 ADWG was significantly higher than G4 during both periods and significantly higher than G2 during the first 7 days post-weaning. Incidence of systemic ARs in G2 and G4 was 0.03% and 0.32%, respectively; none were recorded in G1 and G3. Replacing the usual intramuscular vaccination with MHYOSPHERE® PCV ID results in higher growth performance during the first weeks after weaning with no systemic ARs.

**Supplementary Information:**

The online version contains supplementary material available at 10.1007/s11259-022-09971-y.

## Introduction

Porcine respiratory disease complex (PRDC) causes devastating economic losses to the swine industry due to reduced performance of the pigs, including a decreased growth rate and an increased feed conversion ratio, as well as an increase in the use of antimicrobials, mortality, and treatment costs (Ames 1999; Maes et al. 2003; Yang et al. 2020). As it is considered a major health concern, the pig industry is increasingly interested in the elimination of the pathogens causing PRDC from infected herds or production systems using, among others, vaccination strategies. However, the handling, restrain, fear, and pain associated with vaccination procedures induce physical stress, impacting animal welfare (Hemsworth and Coleman 2010).

PRDC is a multifactorial disease resulting from the interaction of different infectious agents (viruses, mycoplasmas, and bacteria), management and environmental conditions, and host factors (Pallarés et al. 2021). *M. hyopneumoniae* (*Mhyo*) and Porcine circovirus type 2 (PCV2) are among the main causes of PRDC, of which *Mhyo* is a primary causing agent and PCV2 is considered secondary (Segalés et al. 2013)*.* In the field, swine practitioners and producers use vaccination rather than antibiotic treatment and prefer to use single-dose vaccines against *Mhyo* and PCV2 to efficiently control PRDC (Hoelzer et al. 2018; Consortium members of work package 2 led by Andrea Ladinig, University of Veterinary Medicine, Vienna 2020; Yang et al. 2020). Commercially available vaccines against PCV2 use inactivated PCV1-2 chimera virus, inactivated whole virus, or recombinant capsid protein encoded by ORF2 adjuvanted with aqueous polymer (carbomer), D1-α-tocopherol plus liquid paraffin, and light paraffin oil (Chae 2012). Those against *Mhyo* infections are mostly composed of inactivated whole cells of different *Mhyo* strains and adjuvants, such as aluminum hydroxide, carbopol, mineral oil, and biodegradable oil (Opriessnig et al. 2007; Matthijs et al. 2019). *Mhyo* and PCV2 vaccines may be combined in the field before their intramuscular administration, a process that might result in increased vaccination costs due to increased preparation times and possible vaccine mixing errors.

In addition to the inconveniences caused by the need of two separate vaccines to protect against *Mhyo* and PCV2 intramuscular injections using needles are considered painful and induce stress to piglets (Scollo et al. 2020; Temple et al. 2020; Dalmau et al. 2021). Furthermore, administration of two vaccines in association may cause increased pain. However, the use of needle-free devices for intradermal vaccination reduces vaccination-associated pain, resulting in decreased behavioral aversive responses and stress compared to conventional intramuscular injections, increasing animal welfare (Scollo et al. 2020; Temple et al. 2020; Dalmau et al. 2021). In this regard, the avoidance of invasive, stressful procedures has been associated with increased body weight gain (Morgan et al. 2019).

Among the different marketed vaccines, MHYOSPHERE® PCV ID consists of the inactivated recombinant *M. hyopneumoniae*^cpPCV2^ strain Nexhyon expressing the PCV2 capsid protein, forming a single active substance. This is the first vaccine administered intradermally using a needle-free device that protects from both agents simultaneously. In previous studies, MHYOSPHERE® PCV ID has been proven to induce onset of immunity for *Mhyo* and PCV2 as early as 3 and 2 weeks post-vaccination, respectively, and provide protection until at least 22–23 weeks post-vaccination, with a beneficial effect on growth performance, reduced culling rate, and loss of daily weight gain caused by *Mhyo* and/or PCV2-related diseases (Montané et al. 2021a, 2021b; Puig et al. 2021; Simon-Grifé et al. 2021). Furthermore, it reduces lung lesions caused by *Mhyo* infections and, for PCV2-associated diseases, it reduces viremia and virus shedding (nasal and fecal), including absolute values and proportion of viremic pigs and those shedding the virus (Montané et al. 2021a, 2021b; Puig et al. 2021; Simon-Grifé et al. 2021). Moreover, PCV2 genotype (a, b and d) cross-protection was demonstrated under field conditions (Montané et al. 2021b).

Despite the large number of studies assessing the efficacy of vaccination against *Mhyo* and PCV2, face-to-face safety comparisons of vaccine options under field conditions are missing, and the effects of the novel vaccine formulation and method of administration of MHYOSPHERE® PCV ID on the initial development of piglets compared to those of other commercially available vaccines remain unassessed. Given the stress associated with weaning and its detrimental effects on piglets’ health, it is essential to ensure safety and minimize stress during this period (Campbell et al. 2013). This study aimed to compare the effects of MHYOSPHERE® PCV ID when administered at weaning (approximately 3 weeks of age) with those of three different commercially available associations of vaccines under field conditions, using growth performance during the nursery period and incidence of adverse reactions after vaccine administration as welfare and safety parameters.

## Materials and methods

### Study animals and experimental design

This was a randomized, blinded, controlled field trial including three-week-old clinically healthy piglets. The study was conducted in one commercial pig farm located in Spain with historical records of clinical and subclinical *Mhyo* and/or PCV2-related disease. Specifically, symptoms of PCV2 infection and the typical lung lesions caused by *Mhyo* infection had been reported in the past, and routine vaccination against PCV2 and *Mhyo* was implemented before this study started. Since the vaccination program started, both infections have been considered controlled. This farm has a continuous flow of piglets into the weaning unit in batches of 400–800 animals. Upon entrance to the nursery period at 21 days of age, piglets in each batch were randomized at 1:1 ratio based on stratification by covariates of interest (sex, genetics, and weight) to receive two treatments: test vaccine (Group 1 [G1]) or comparator vaccine (Groups 2, 3, or 4 [G2, G3, or G4, respectively]). Animals received the test vaccine in a single intradermal administration and each one of the three comparators in two different intramuscular injections in the neck area (one against *Mhyo* and one against PCV2).

### Study products

Industrial standard batches of the test vaccine MHYOSPHERE® PCV ID (HIPRA, Spain), G1; and the comparators Ingelvac Circoflex® (Boehringer Ingelheim, Germany) + Hyogen® (CEVA, France), G2; Porcilis® PCV (MSD, USA) + M + PAC® (MSD, USA), G3; and Porcilis® PCV + Hyogen®, G4, were administered at the doses indicated in the summary of product characteristics. The four vaccination approaches corresponding to each study group are described in Table 1. The rationale for the comparators’ choice was guided by the farmer’s and farm veterinarian’s preferences and interests based on the previous farm experience with PCV2 and *Mhyo* vaccines and was a sponsor-farmer shared decision. These comparator vaccines are the most used vaccines in the field and are considered as references from the veterinarians’ point of view. Furthermore, the PCV2 and *Mhyo* vaccine associations assessed are the most frequent in the global market. In this regard, Circoflex® is the reference and leader PCV2 vaccine on the market, and Hyogen® is considered the reference *Mhyo* vaccine on the market in Spain. The details of the study timeline and animal batches are provided in Fig. S1 (Supplementary material).

**Table 1:** Characteristics of the vaccines and associations administered in this study

	Test vaccine	Comparators		
	Group 1	Group 2 (Association)	Group 3 (Association)	Group 4 (Association)
Product/s name/s	MHYOSPHERE® PCV ID	Ingelvac Circoflex® + Hyogen®	Porcilis® PCV + M + PAC®	Porcilis® PCV + Hyogen®
Composition	Inactivated recombinant *Mycoplasma hyopneumoniae*^cpPCV2^, strain Nexhyon	Porcine circovirus type 2 ORF2 protein + Inactivated *M. hyopneumoniae* strain 2940	Porcine circovirus type 2 ORF2 subunit antigen + Inactivated *M. hyopneumoniae* strain 25,934	Porcine circovirus type 2 ORF2 subunit antigen + Inactivated *M. hyopneumoniae* strain 2940
Dose	0.2 mL	1 mL + 2 mL	2 mL + 2 mL	2 mL + 2 mL
Administration route	Intradermal (needle-free device)	Intramuscular	Intramuscular	Intramuscular
Presentation	Emulsion for injection	Suspension for injection + emulsion for injection	Emulsion for injection	Emulsion for injection

### Clinical safety evaluation

The incidence of adverse reactions as well as the effect of vaccination on growth performance, used as a surrogate measure of animal welfare, were monitored during the study to evaluate the clinical safety of the test vaccine compared to the different vaccine associations. Different studies support a relationship between animal welfare and growth performance. In pigs, feeding patterns may be used as welfare indicators, and different stressors have been shown to impact growth performance (Martínez-Miró et al. 2016; Bus et al. 2021). Furthermore, a recent study showed that intramuscular vaccination was associated with lethargy and decreased feed intake after weaning, compared to intradermal vaccination, resulting in decreased growth performance during the nursery period (Bruna Ferrandin et al. 2022). Site staff responsible for safety evaluation observed the piglets immediately after vaccination and monitored them for two to four hours to record noticeable adverse reactions. Adverse systemic reactions considered were those not requiring pig handling, such as anaphylactic shock and vomiting, to preserve the field conditions and avoid additional manipulations causing piglet stress. Likewise, local adverse reactions, which may appear several hours after vaccination, were not monitored to minimize piglet manipulation. The live weight of pigs in each barnyard was measured at several time points between vaccination and the end of the nursery period (d35-d42 after vaccination). The duration of the nursery period ranged between 35 and 42 days for different batches. The average daily weight gain (ADWG; g/day) was calculated and analyzed over different periods: a) during the first 7 days and b) between the vaccination day (d0) and end of the nursery period. ADWG during the different periods was calculated as the difference between the starting and final weight divided by the duration of the stage. Data for dead or removed pigs were not excluded from the calculation due to logistic reasons.

Additional variables considered were pig’s batch (1 to 12), genetics (F2 and F7), sex, pen (A, B, and C), weight at entrance, which was categorized into 4 groups (< 4.78 kg, 4.78–5.6 kg, > 5.6–6.4 kg, and > 6.4 kg), and diarrhea (diarrhea during the first week and no diarrhea).

### Statistical analysis

Categorical variables were presented as frequencies and percentages, and quantitative variables as the mean and standard deviation (SD) and the median and minimum/maximum. Descriptive statistics of the animals’ characteristics were applied to productive and clinical variables as required.

A multivariable linear regression method was applied to compare growth performance between vaccine groups. The initial model also included genetics, sex, pens, weight at entrance in the study, days of stay and diarrhea as fixed effects, and batch as random effect. All variables that retained a significant independent association (*p* < 0.05) were included in the final model, resulting in a final model considering genetic subgroup, weight at entrance, and batch as covariates. All statistical analyses were performed using R software (version 4.0).

## Results

### Study population

**Fig. 1:**
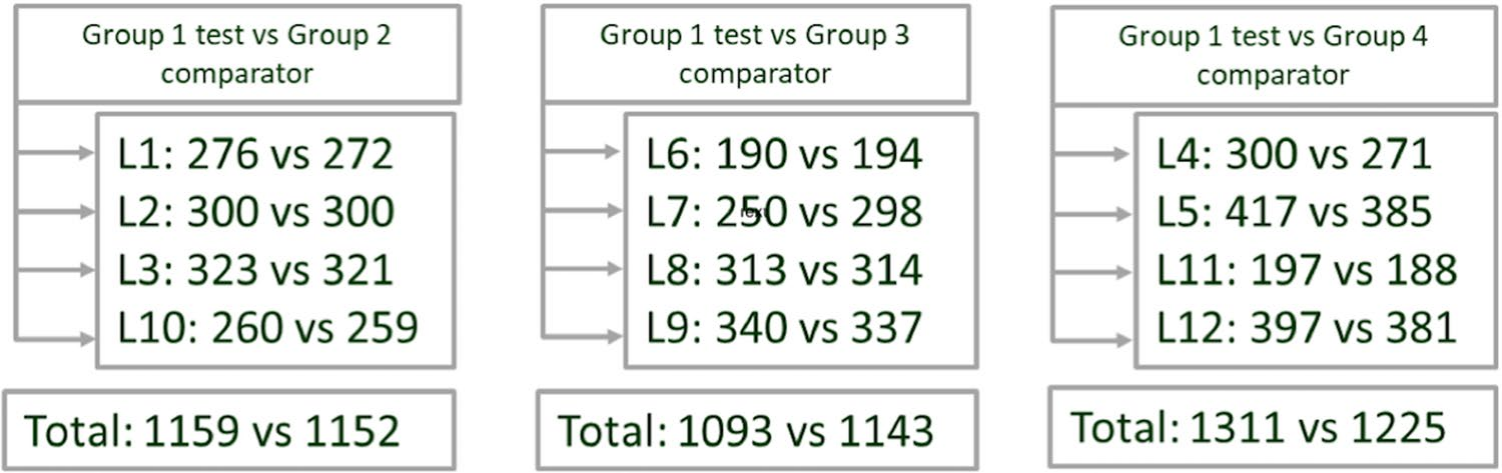
Distribution of piglets based on treatment comparators and batch

A total of 7072 21-day-old piglets (balanced number of males and females) from 12 consecutive weekly batches entering the nursery were randomly distributed into four groups: 3552 received the test vaccine MHYOSPHERE® PCV ID (G1); 1152 received the G2 control vaccine; 1143 received the G3 control vaccine; and 1225 received the G4 control vaccine (Fig. 1). Mean (SD) body weight at entrance was 5.57 (1.17) kg; most animals (88.6%) had the F2 genotype and the remaining 11.4% the F7 genotype. The distribution of piglets in weight-at-entrance categories according to treatment group and comparison is shown in Fig. S2 (Supplementary material).

### Growth performance

**Fig. 2:**
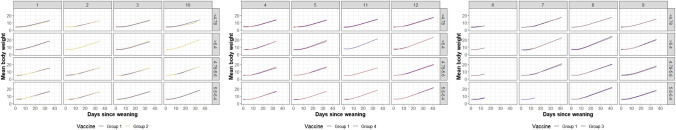
Growth performance of piglets of the F2 genetic subgroup. Growth curves, represented as mean weight increase over time, of the different batches based on weight at entrance (< 4.78, 4.78–5.6, 5.6–6.4, and > 6.4 kg) and treatment are shown

The multivariable regression analysis indicated that sex, pens, days of stay at nursery (35 or 42), and diarrhea were not associated with growth performance (*p* > 0.05). The genetic subgroup, weight at entrance, and batch variables were associated with growth performance and were therefore included in the final model (*p* < 0.05). The F2 genetic subgroup was the most frequent in this study (88.6%) and showed significantly increased ADWG at the end of the nursery period, compared to F7 genetic group (Fig. S3). Likewise, increased weight at entrance was associated with higher growth performance (Fig. S4). The variability in growth performance among batches was very high and was considered a random effect. Figure 2 displays analysis of growth performance of the F2 genetic subgroup according to batch and treatment group.

Results from the regression model, summarized in Table 2, showed that growth performance measured as ADWG (g/day) was higher in G1 than in G2, G3, and G4 during the first 7 days after vaccination, being the differences statistically significant when compared with G2 and G4. Similarly, growth performance at the end of the nursery period was higher in G1 than in G2, G3, and G4, being the differences statistically significant when compared with G4. Overall, ADWG for G1 was higher than for the other vaccine associations in both study periods.

**Table 2:** Estimated differences in growth performance (measured as average daily weight gain) of G1 (test vaccine) compared to G2, G3, and G4 (control vaccine associations) at the indicated study periods

Differences in ADWG	Group 2 Ingelvac Circoflex® + Hyogen®	Group 3 Porcilis® PCV + M + PAC®	Group 4 Porcilis® PCV + Hyogen®
0–7 days (g/day)	−10.92	−3.04	−20.08
*P* value vs. Group 1	**0.018**	0.522	**< 0.001**
0-End nursery (g/day)	−0.65	−4.06	−9.58
*P* value vs. Group 1	0.869	0.445	**0.017**

*ADWG* average daily weight gain

*P*-values in bold font denote statistically significant differences (*p* <0.05) between G1 and the other vaccine groups

### Incidence of adverse reactions

Five animals from two different study groups experienced post-vaccination systemic reactions, one animal (0.03%) in G2 and 4 (0.32%) in G4, consisting of anaphylactic shock and vomiting. Specifically, one anaphylactic shock of mild severity was observed in one animal from batch 1 in G2 (0.37%). Two anaphylactic shocks were recorded in batch 4 of G4 (0.74%). Finally, one anaphylactic shock and one episode of vomiting were observed in batch 12 of G4 (0.52%). No adverse reaction was observed in G1 and G3.

## Discussion

The present study assessed the effects of the vaccine MHYOSPHERE® PCV ID compared to those of three associations of commercially available vaccines against *Mhyo* and PCV2 infections under field conditions during the nursery period, by analyzing growth performance during the nursery period and incidence of adverse reactions after vaccination. Results from this study showed that piglets receiving the test vaccine (G1) had significantly higher growth performance compared to G2 and G4 during the first 7 days after vaccination, as well as significantly higher growth performance compared to G4 during the whole nursery period, without post-vaccination adverse reactions related to the vaccine.

Performance of pigs from farrowing to slaughter is the result of a complex interaction of factors, of which those in the early stages of a pig’s life are likely to affect lifetime performance (Kats et al. 1992; Douglas et al. 2014). The weaning process at 3–4 weeks of age is one of the most stressful events in pigs’ lives, and can contribute to intestinal and immune system dysfunctions, resulting in reduced pig health, growth, and feed intake, particularly during the first week after weaning. During this time, pigs are subject to a number of stressors, such as an abrupt separation from the sow, transportation and handling stress, diet change, social hierarchy stress, co-mingling with pigs from other litters, a different physical environment (room, building, farm, water supply, etc.), and increased exposure to pathogens and dietary and environmental antigens (Campbell et al. 2013). In general, pigs lose about 100–250 g of body weight (BW) on the first day after weaning, regardless of weaning age, and recover this loss by about 4 days post-weaning (Le Dividich and Sève 2000).

BW variability hinders farm efficiency and occupation time, mainly regarding the growing-finishing facilities, and constitutes a limiting factor for the current all-in-all-out swine production systems. López-Vergé et al. showed a tight correlation between body weight at weaning and subsequent phases of production, preventing lighter piglets from catching up with their weightier counterparts. As a result, from weaning onwards, piglets’ categories are maintained (López-Vergé et al. 2018). *M. hyopneumoniae*^*c*pPCV2^ lacked any detrimental effect on BW. On the contrary, showed increased post-weaning growth performance compared to other vaccination alternatives. These results show that MHYOSPHERE® PCV ID is a good option to ensure normal piglet development and performance later in life.

The unique formulation and new active substance of *M. hyopneumoniae*^*c*pPCV2^ allows to reduce the administration volume to 0.2 mL, enabling the administration of the active substance by intradermal route using a needle-free device. Even though this study did not compare administration routes (intradermal vs. intramuscular administration), associations between intradermal administrations and increased animal welfare have been previously reported (Scollo et al. 2020; Temple et al. 2020). Temple et al. showed that piglets vaccinated intradermally (ID) had lower blood C-reactive protein and blood haptoglobin levels at 28 h post-vaccination compared to piglets vaccinated intramuscularly (IM), preventing the acute phase response and muscular damage associated with intramuscular injections (Temple et al. 2020). Sánchez-Matamoros et al. postulated that the extended times needed to cross the raceway and higher vocalization (presence and power) showed that IM injection was more aversive for piglets than ID injection (Sánchez-Matamoros et al. 2021). In the present study, pigs receiving the control vaccine associations received two intramuscular injections. In this context, results from this study showing increased growth performance in pigs vaccinated with MHYOSPHERE® PCV ID may reflect a good health state (including the absence of negative experience, such as pain), suggesting a potential contribution to increased animal welfare. In this regard, a recent study showed that intramuscular vaccination was associated with lethargy and decreased feed intake after weaning, compared to intradermal vaccination, resulting in decreased growth performance during the nursery period (Bruna Ferrandin et al. 2022). Aside from the increased growth performance, intradermal vaccination using a needle-free technology has been shown to reduce transmission of PRRSV and other diseases, further contributing to controlling disease dissemination (Otake et al. 2002; Baker et al. 2012; Nilubol et al. 2022).

The results of this study should be interpreted in the context of limitations associated with its field setting. This study was conducted on one commercial farm where individual animals are not usually traced. For this reason, unlike more controlled settings, some data may have remained unrecorded, including mortality data and individual weight of dead pigs, which could not be eliminated from the analysis. Even though specific conditions of the farm might have influenced this study’s results, the farm’s clinical history and characteristics were those of any commercial farm, and we believe that the results comparing the different vaccines are likely generalizable to other farms. Given the nature of this field study aiming to capture the impact of a safe intradermal vaccine on production parameters under field conditions, additional handling of piglets after vaccination, representing an additional source of stress, was considered undesirable. Consequently, additional safety parameters such as rectal temperature and local reactions at the injection site remained unassessed. Nevertheless, the results of this study allowed to capture and compare the performance of MHYOSPHERE® PCV ID with that of three different *Mhyo* and PCV2 vaccine associations, measured as piglet growth under field conditions during the stressful weaning process, providing valuable information for the swine industry.

In conclusion, all the vaccination strategies assessed in this study were safe and did not induce any undesirable severe adverse reactions. Comparison of growth performance of piglets receiving the different vaccines showed that a single dose of MHYOSPHERE® PCV ID vaccine administered intradermally with a needle-free injector resulted in increased growth in the first seven days post-vaccination compared to vaccination comprising associations of single *Mhyo* and PCV2 vaccines. In addition, the ready-to-use *M. hyopneumoniae*^*c*pPCV2^ formulation in a single preparation and with only one active substance eliminates the disadvantages associated with the need to administer *Mhyo* and PCV2 vaccines in two independent IM injections. Given the increased growth performance and low adverse reactions shown in this study and the advantages of intradermal vaccination, the MHYOSPHERE® PCV ID vaccine stands out as a unique user-friendly and safe product for the porcine industry and, as shown in previous studies, contributes to controlling *Mhyo* and PCV2-associated diseases.

## Supplementary Information

ESM 1 (DOCX 3.59 MB)

## Data Availability

Data from this study are available upon reasonable request from the corresponding author.
