# Supplementary material for: Comparison of effects of a single dose of MHYOSPHERE® PCV ID with three commercial porcine vaccine associations against Mycoplasma hyopneumoniae (Mhyo) and porcine circovirus type 2 (PCV2) on piglet growth during the nursery period under field conditions
Source: Vet Res Commun. 2022 Jul 13;46(4):1167–73. doi: 10.1007/s11259-022-09971-y (PMC9684222; doi:10.1007/s11259-022-09971-y)
Supplement: Supplementary file 1 — (DOCX 3.59 MB) [file 11259_2022_9971_MOESM1_ESM.docx]

**Supplementary Materials for Submission by Puig et al. “Comparison of effects of a Single Dose of MHYOSPHERE^®^ PCV ID with Three Commercial Porcine Vaccine Associations against *Mycoplasma hyopneumoniae (Mhyo)* and Porcine Circovirus Type 2 (PCV2) on Piglet Growth during the Nursery Period under Field Conditions.”**

**Supplementary Figures**


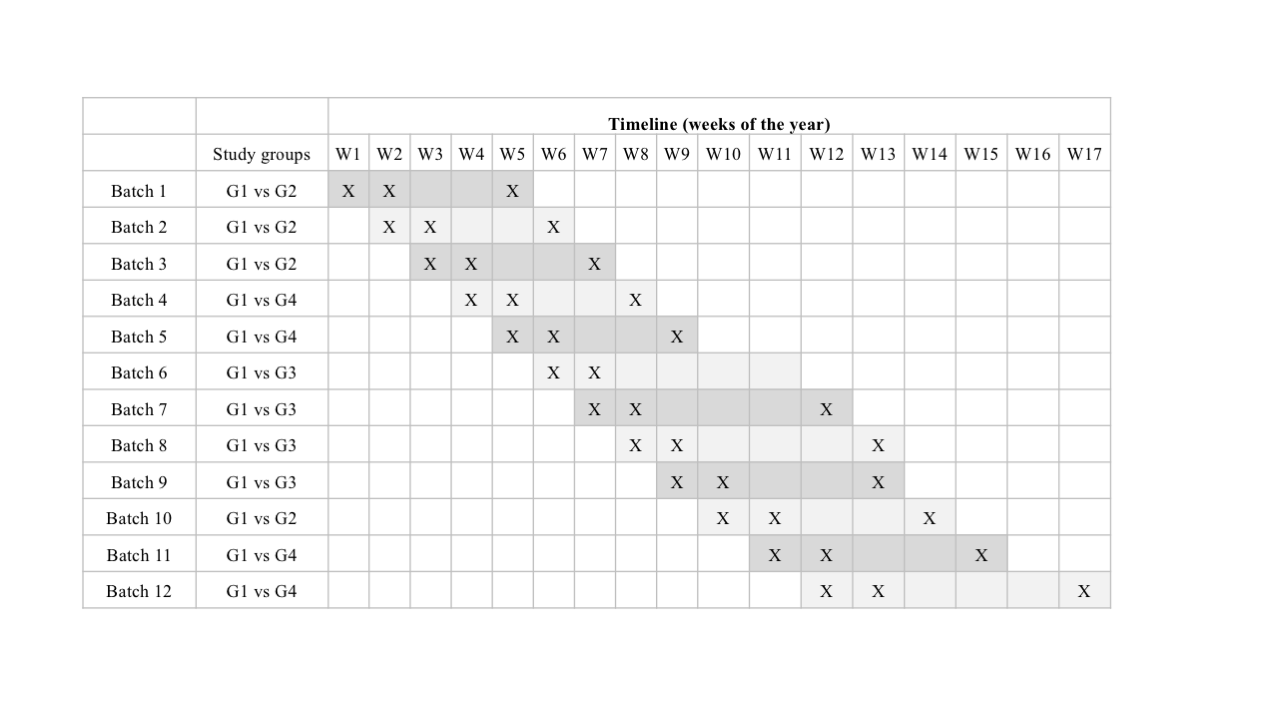
**Figure S1**. Study timeline, batches, and groups.

**
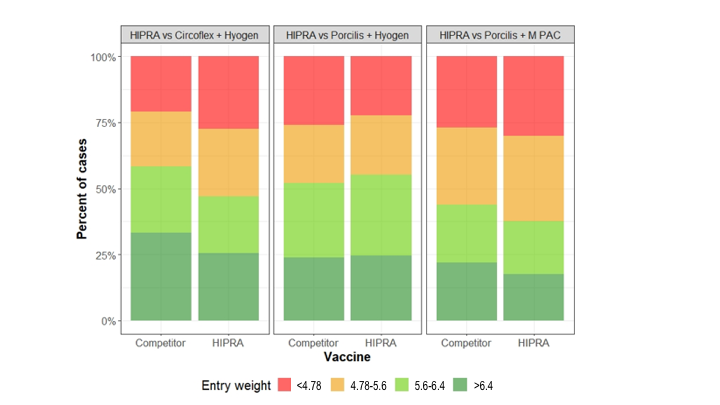
**

**Figure S2.** Distribution of piglets in different weight categories at entrance (i.e., < 4.78 kg, 4.78–5.6 kg, 5.6–6.4 kg, and > 6.4 kg) according to treatment group and comparison.

**
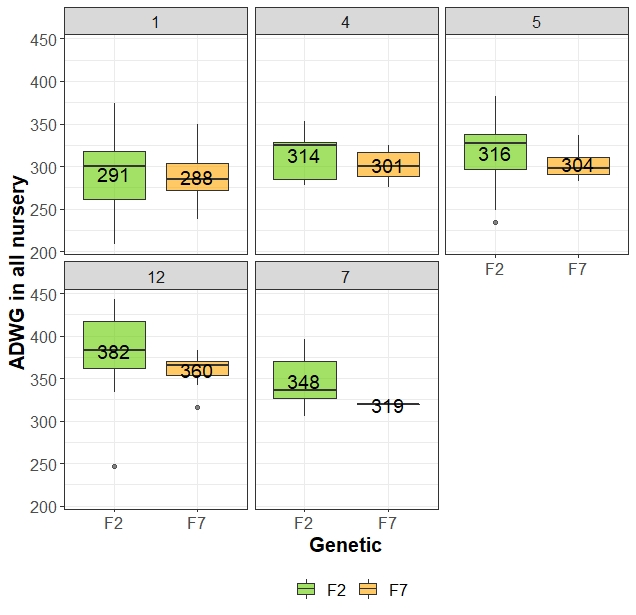
**

**Figure S3.** Box-plot of average daily weight gain (g/day) at the end of stay of batches 1, 4, 5, 12, and 7 based on genetic background (F2 vs. F7). The box represents the interquartile range, the whiskers the minimum and maximum, and the median is represented by a horizontal line inside the box. ADWG, average daily weight gain.


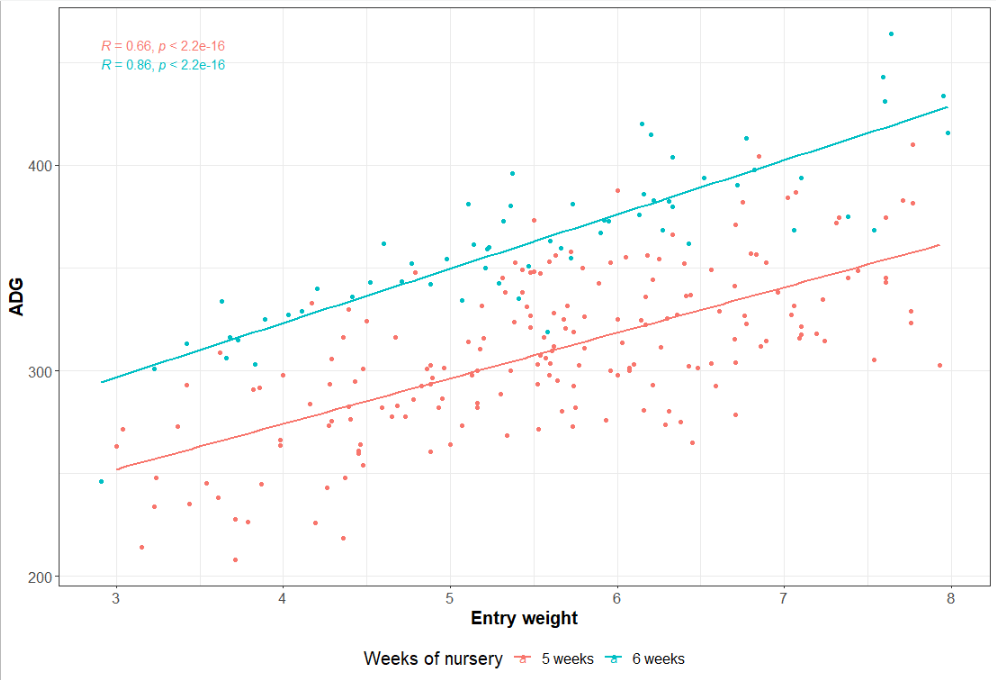


**Figure S4**. Correlations between weight at study entrance and growth performance, measured as average daily weight gain (g/day). ADWG, average daily weight gain. Correlation values correspond to the Pearson correlation test.
